# A New Pallet-Positioning Method Based on a Lightweight Component Segmentation Network for AGV Toward Intelligent Warehousing

**DOI:** 10.3390/s25072333

**Published:** 2025-04-07

**Authors:** Bin Wu, Shijie Wang, Yi Lu, Yang Yi, Di Jiang, Mengmeng Qiao

**Affiliations:** College of Mechanical and Electronic Engineering, Nanjing Forestry University, Nanjing 210037, China; wangshijie@njfu.edu.cn (S.W.); yiyang_njfu@163.com (Y.Y.); jiangdi@njfu.edu.cn (D.J.); qiaomengmeng2024@njfu.edu.cn (M.Q.)

**Keywords:** pallet positioning, component segmentation, attention mechanism, generalization capability, deep learning

## Abstract

In human–robot hybrid intelligent warehouses, pallets often come in various shapes and sizes, posing challenges for AGVs to automate pallet picking. This, in turn, reduces the overall operational efficiency of the warehouse. To address this issue, this paper proposes a lightweight component segmentation network using a dual-attention mechanism to achieve precise segmentation of the pallet’s stringer board and accurate localization of the pallet slots. To overcome the challenge of redundant computations in existing semantic segmentation models, which are unable to balance spatial details and high-level semantic information, this network utilizes a dual-branch attention mechanism within an encoder–decoder architecture to effectively capture spatial details. On this basis, a residual structure is introduced to reduce redundant network parameters, addressing issues like vanishing and exploding gradients during training. Due to the lack of a public pallet image segmentation dataset, the network was tested using a custom-made dataset. The results show that by extracting intermediate-, low-, and high-level features from dual-branch input images and integrating them to construct multi-scale images, precise segmentation of various types of pallets can be achieved with limited annotated images. Furthermore, to comprehensively evaluate the model’s robustness, additional pallet localization experiments were conducted under varying illumination conditions and background noise levels. The results demonstrate that the proposed method can effectively identify and locate multi-category pallet targets while maintaining high segmentation accuracy under different lighting conditions and background interferences, verifying the model’s robustness in complex warehousing environments. Compared to the traditional model, the proposed model in this paper achieves a 10.41% improvement in accuracy and a 32.8% increase in image processing speed. The segmentation network we proposed is used for pallet-positioning experiments and has achieved good positioning results in pallet images taken from different distances and angles.

## 1. Introduction

The intelligent warehouse is a critical component of the supply chain in intelligent manufacturing, which cannot only reduce labor costs, minimize material loss, and enhance production safety, but also serve as a crucial guarantee for achieving intelligent production of products [1]. As the demand for global industrial upgrading continues to increase, many warehouses are facing the need for intelligent transformation and upgrades [2]. Using automated guided vehicle (AGV) forklifts in conjunction with pallets for automated material handling and storage is the main form of operation in current intelligent warehouses [3]. However, due to cost and geographical constraints, most intelligent warehouses have been upgraded from traditional manual warehouses. Consequently, many warehouses still operate in a human–robot hybrid mode, leading to random placement and orientation of pallets within the warehouse, which poses challenges for AGVs in automatically picking up and placing goods [4].

To accurately locate the position and posture of the pallet is a prerequisite for AGV to pick and place goods. Currently, the methods commonly used in the industry are QR code-assisted positioning and LiDAR positioning [5]. The QR code-based positioning method can effectively determine the type of goods and the position of the pallet [6]. However, in a human–robot hybrid warehouse, pallet placement angles and positions are not only random, but the types of pallets are also diverse. On the one hand, attaching QR codes consumes a large amount of labor and material costs. On the other hand, QR codes cannot provide information on the angle deviation of the pallet. When the surface of the pallet with the QR code is not facing the AGV directly, the AGV cannot even recognize and locate the pallet position [7]. For the LiDAR positioning method, apart from its high cost, there is also the challenge that when pallet types are inconsistent and the sizes of the insertion holes vary, the forklift finds it difficult to accurately locate the pallet slots for picking and placing goods [8]. Therefore, how to quickly and cost-effectively locate randomly placed pallets and achieve precise insertion and retrieval of goods by AGV forklifts are urgent problems that need to be solved in the field of smart warehousing [9].

To achieve the lowest cost, the image-based pallet location and recognition method has become a research hotspot in the field of intelligent warehouses. A pallet pose recognition algorithm based on an RGB-D camera was developed [10]. Image-based pallet-positioning methods primarily analyze image characteristics to detect pallets by identifying distinctive features within an image. Methods that utilize geometric [11] and color features [12] are proposed for pallet detection; these approaches employ mathematical morphology to define the edge points and corner points of the pallets. However, relying on color or geometric thresholds to distinguish pallets from backgrounds is unreliable, as actual warehouse environments often contain diverse types and colors of both pallets and backgrounds. The ROBOLIFT system detects pallets using region-growing algorithms [13]. However, this system fails to maintain positioning accuracy and reliability when dealing with different types of pallets or random placement angles. Subsequent research studies have proposed detecting the edges where pallets contact the ground directly and estimating their orientation; however, this method performs poorly in cases where edge features are not clearly defined.

An improved UPerNet network was developed to achieve pallet recognition in complex warehouse environments [14]. Although the researchers optimized the network to segment pallets in complex scene images, the algorithm is still limited to overall pallet recognition. Precise insertion of the forklift into the pallet after localization requires additional methods. Precise insertion of the pallet by an AGV forklift after localization still requires additional methods. For example, images and point clouds captured by an RGB-D camera were utilized to localize the pallet slots of the pallet [15]. First, the front face of the pallet was segmented using the YOLOv5 network. Subsequently, depth information and a point cloud segmentation algorithm were employed to establish specific segmentation criteria for determining the center coordinates of the pallet slots. This information was subsequently used to guide the forklift in transporting the pallet. This method is mainly suitable for situations where the forklift is facing the pallet directly and requires prior knowledge of the pallet’s features and dimensions. When the angle between the pallet and the AGV is random or the type of pallet changes, this method may produce significant errors. On the other hand, in human–robot hybrid warehouses, AGVs need to quickly assess the posture of target goods and free up resources to respond to the sudden appearance of people or other vehicles to prevent safety accidents. Currently, there are few reports on the optimization of response time and computational resources for pallet localization detection networks.

In human–robot hybrid intelligent warehouses, how to achieve pallet precise positioning through the obtained visual information is an urgent problem that needs to be solved in the field of intelligent warehousing [16]. Among these challenges, the precise segmentation of the target pallet stringer board is crucial for successfully completing pallet positioning for AGVs. To achieve accurate and efficient localization of pallet posture and slots, while ensuring the network has a degree of generalization ability, this work presents a lightweight component segmentation network based on a dual-attention mechanism (LCS-Net). The network integrates dual-attention mechanisms to enhance segmentation accuracy and optimizes the backbone to improve computational efficiency. Comprehensive experimental validation demonstrates the model’s ability to generalize across various pallet types. These innovations enable LCS-Net to improve segmentation accuracy and computational speed, addressing critical issues such as pallet positioning and slot identification in human–robot hybrid intelligent warehouse environments. Experimental results show that LCS-Net excels at accurately locating pallets of different types, meeting the real-time demands of complex warehouse environments. By combining multi-scale semantic information and focusing on the weights of different channels and spatial positions, the network improves component segmentation performance. Summing the outputs of the two attention modules further enhances feature representation. Additionally, by adaptively learning feature information and capturing rich contextual dependencies, the network can effectively tackle scene segmentation tasks, further improving segmentation performance.

## 2. Related Works

In human–robot hybrid intelligent warehouses, where pallet position deviations are significant, it is necessary to obtain precise location information of the target pallet through supplementary visual information. How to utilize this visual information to detect and accurately locate pallets is an urgent issue that needs to be addressed in the field of smart warehousing. In many applications, such as image recognition, target tracking, and robot navigation, image segmentation is a necessary prerequisite. Currently, there are still some urgent issues to be resolved in the application of image segmentation.

### 2.1. Blurry Edge Segmentation and Slow Segmentation Speed

In recent years, the development of deep learning techniques has brought new breakthroughs to image segmentation [17], and many studies have evaluated the performance of these algorithms. Among them, the FCN is used to extract buildings from high-resolution images [18], but FCN only uses high-level feature maps for pixel classification and low-level feature maps are discarded, resulting in a limited ability to process small targets. To address this issue, some semantic segmentation models that focus on handling low-level feature points have emerged, such as U-Net [19] and SegNet [20]. These semantic segmentation models adopt an encoder–decoder architecture, where the encoding region is used for feature extraction and the decoding region restores the extracted feature map to the original image scale. During the process of restoring feature maps, the decoding region fuses with the low-level feature maps of the encoding region to obtain feature information at different levels and improve segmentation accuracy. However, the high-level semantic features lack sufficient detailed information, resulting in poor segmentation results. In order to obtain advanced semantic information to solve the problem of classification errors, ResNet [21] gradually extracts advanced semantic information by continuously deepening the network. Later, the DeepLabv3+ network became popular [22]. It combines semantic information from different scales of receptive fields to improve the classification ability of the model. However, such networks rely on downsampling to obtain advanced semantic information, ignoring spatial details, and having problems such as blurred segmentation edge detection. Obtaining spatial detail information and high-level semantic information is always contradictory, but for solving the problem of inaccurate edge contour segmentation, the spatial detail information of images is equally important. In order to address related defects, dual-branch networks have been proposed [23], which separately extract processing spatial detail information through new branches and design feature fusion modules to fuse advanced semantic information and spatial detail information. This is very effective for segmenting edge contours, but it also brings about greater computational complexity. Solving the problem of segmentation accuracy types often requires more computational resources and longer network training times. Ref. [24] developed a preliminary method for image segmentation by introducing the convolutional block attention module (CBAM) into DeepLabv3+, which improved the original network’s shortcomings in rough edge target segmentation. The authors applied the improved network to autonomous driving semantic segmentation scenarios, but it still failed to solve the problem of slow network training speeds. Ref. [25] introduced depthwise separable convolution instead of ordinary dilated convolution in hetero receptive field splicing ASPP to accelerate model training speed. However, this improved network primarily addresses the weak detail representation ability of DeepLabv3+, which suffers from mis-segmentation issues. It still has shortcomings in real-time engineering applications.

### 2.2. Small-Sample Semantic Segmentation

With the rapid development of deep learning technology, breakthrough progress has been made in semantic segmentation methods using deep learning. However, the segmentation performance of such methods overly relies on a large amount of annotated training data. In addition, the high cost of per-pixel annotation greatly limits the development of image semantic segmentation, which is also unrealistic in some practical applications [26]. The small-sample semantic segmentation method mainly adopts a dual-branch network structure; it uses techniques [27] to extract the middle-, low-, and high-level features from the dual-branch input image, and then fuses and constructs multi-scale features. Based on this, an iterative optimization few-shot semantic segmentation model is proposed, and the efficiency of the proposed model is verified on the PASCAL and COCO datasets.

The quality of deep learning models depends on the size and quality of the dataset. The data in the warehousing field have problems such as a limited number of annotations, uneven sample distribution, and common data missing phenomena. Therefore, it is necessary to apply small-sample semantic segmentation research to the field of warehousing.

### 2.3. Generalization of the Segmentation Model

In the fields of autonomous driving and smart healthcare, automatic image segmentation has been extensively studied. The most advanced image segmentation models are typically based on structures such as fully convolutional networks (FCNs) and U-Net. These models have achieved high-precision segmentation results across various automatic segmentation tasks. In the implementation of automatic prediction tasks using deep learning neural networks, excellent performance often relies on the assistance of accurately labeled data for training. Therefore, deep models are data-driven methods that heavily depend on labeled information. In the medical field, researchers have found that although deep learning models demonstrate advanced performance on benchmark data, the lack of multi-center generalization leads to difficulties in applying these methods to real clinical problems [28]. In the field of intelligent warehousing, the types and shapes of pallets that need to be segmented vary widely. Automatic segmentation methods that heavily rely on data labeling often struggle to transfer learned knowledge from one center to another. As a result, models can only rely on their inherent self-awareness and generalization capabilities to handle previously unseen target domain data. This challenge is the primary focus of research on model generalization. Therefore, addressing the model generalization issue in the segmentation of multi-center intelligent warehousing scenarios is a practical problem closely related to computer-aided pallet localization.

In human–robot collaborative scenarios where pallet position deviations are significant, it is necessary to obtain precise location information of the target pallet through supplementary visual information. How to use this visual information to detect and accurately locate pallets is an urgent problem that needs to be solved in the field of smart warehousing. The warehousing field is characterized by complex application scenarios and a wide variety of target objects. The effectiveness of deep learning models depends on the size and quality of the dataset. However, in the warehousing field, the data often suffer from limited labeling, imbalanced sample distribution, and widespread data missing issues. Therefore, further improvements to existing object segmentation models are needed for practical application. To summarize, the following contributions are presented in this paper:

LCS-Net is proposed to address the issues of limited annotation quantity, uneven sample distribution, and common data missing in redundant computing and warehousing due to the inability of existing real-time semantic segmentation network models to balance spatial detail information and advanced semantic information. Key points include the following:

1. Obtaining spatial detail information through a dual-branch attention network and optimizing the weights of deep features to solve the problem of inaccurate pallet edge segmentation.
2. Extracting the middle-, low-, and high-level features of the dual-branch input image and fusing them to construct multi-scale images, which guide precise segmentation of unknown new classes using limited annotated images.
3. Introducing residual structures and inverted residual structures in the backbone network to reduce redundancy in network parameters. During the image generation phase, a noise component is introduced, increasing data diversity and further enhancing the model’s generalization ability.

## 3. Methodology

### 3.1. Using the Segmentation Network to Achieve Pallet Positioning

**Figure 1:**
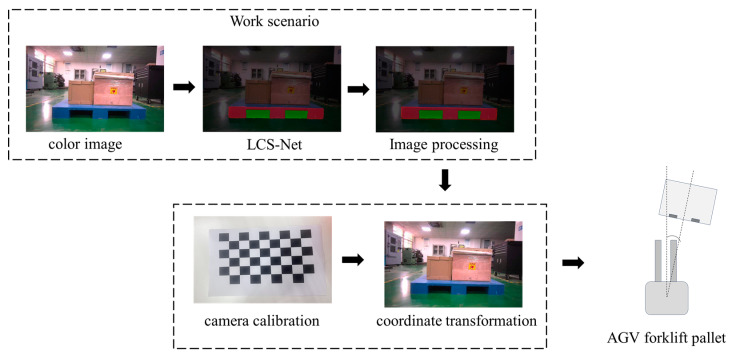
AGV intelligent pallet forking system framework.

The process for pallet positioning is shown in Figure 1. Using the LCS-Net to segment the pallet stringer board, further image processing is performed on the pallet stringer board. To achieve the positioning of the center point of the pallet stringer board, it is necessary to first calibrate the extrinsic parameters of the camera in the warehouse working environment. This will determine the transformation relationship between the pixel coordinate system and the pallet stringer board coordinate system, generating a conversion formula between the two coordinate systems. By processing the image, the minimum enclosing rectangle of the segmented pallet stringer board can be obtained, from which the pixel coordinates of the center of the stringer board are derived. Using the coordinate transformation formula, the three-dimensional coordinates of the center point of the pallet stringer board can be obtained.

### 3.2. Basic Architecture of LCS-Net

**Figure 2:**
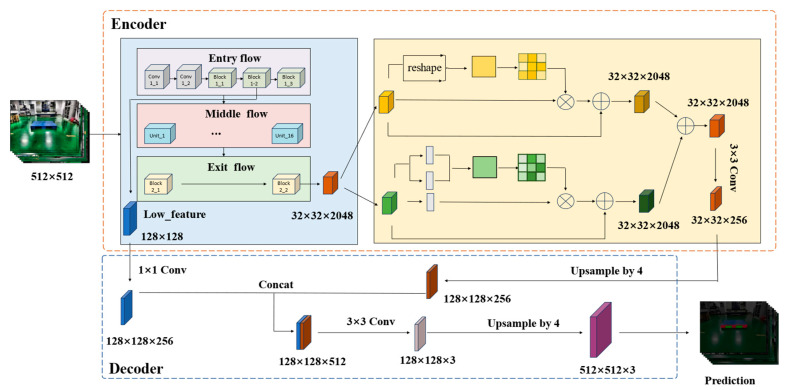
Network structure diagram.

The pallet stringer board is usually the same color as the other surfaces of the pallet, making the boundaries of these surfaces in the image blurry and difficult to distinguish. Therefore, segmenting individual stringer boards is challenging. This phenomenon is particularly noticeable in oblique and isometric views. To enhance edge recognition capabilities and maintain the scalability of network functions, the network proposed in this paper adopts an encoder–decoder structure. LCS-Net is shown in Figure 2. In the encoder backbone network, an improved Xception is used as the feature extractor, and a dual-attention module is used for multi-scale field perception. The position attention module and channel attention module operate in parallel within the dual-attention module. Firstly, the improved backbone network Xception extracts the input image and divides it into two parts: shallow features and deep features. The deep features are downsampled 16 times and then convolved with an expansion rate of 2 by 3 × 3. The encoding region of this article adopts a dual-branch network structure, which processes spatial detail information and high-level semantic information respectively, achieving high-precision and efficient real-time segmentation of pallet components, they are then separately fed into the position attention module and the channel attention module for processing. The position attention module aggregates the features of each position by weighting the sum of all position features, promoting classification accuracy between different local features. The channel attention module allocates appropriate attention weights to each channel based on the interdependence between channel maps, improving segmentation accuracy. Shallow features are downsampled by a factor of four before entering the decoding region of the network.

### 3.3. Optimization of Xception Network

**Figure 3:**
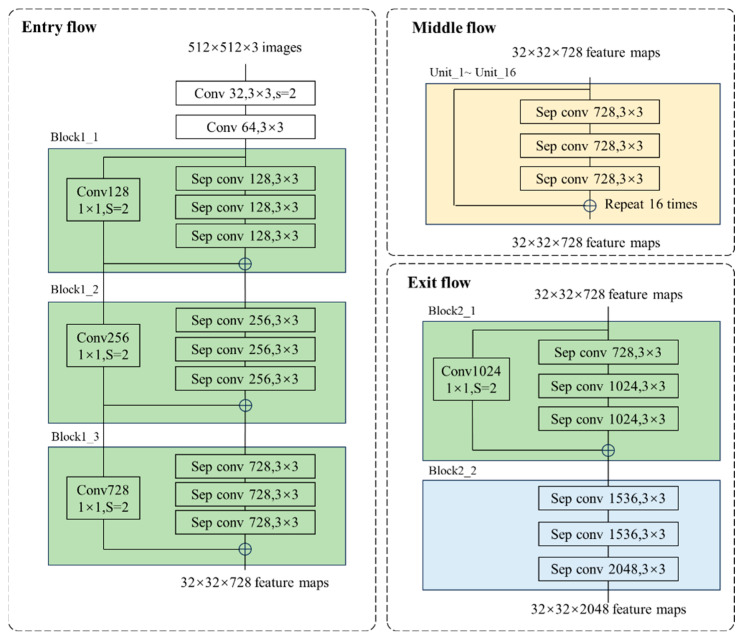
Schematic diagram of improved Xception network structure.

Improving the segmentation accuracy of network models often increases the training time and computational resources of the network. In human–robot hybrid intelligent warehouses, AGVs need to make decisions in the shortest time while minimizing the demand for computational resources. Therefore, while considering segmentation accuracy, the scale and segmentation speed of the model also need to be further improved. This article improves Xception; the improved Xception uses depthwise separable convolutions instead of max pooling layers, allowing the network to extract feature information at any resolution and increase the receptive field. As the neural network deepens, the number of features that the network can extract also increases. However, this also leads to problems such as gradient explosion and vanishing, making it difficult to train the network. Therefore, the improved Xception network adds batch normalization and H-Swish residual structures after each 3 × 3 depth separable convolution. Batch normalization first processes the same batch of data then applies scaling coefficients and offsets to correct it. This stabilizes the data from the preceding layers and reduces the influence of the parameters from the earlier layers on those of the subsequent layers. The input data of the backbone network are sequentially passed through the entry flow layer, middle flow layer, and exit layer to obtain the final output; the network structure is shown in Figure 3.

**Figure 4:**
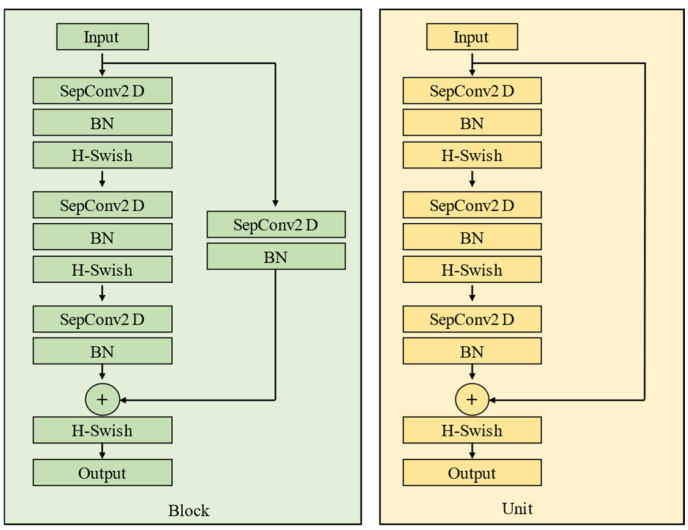
Residual structure with depthwise separable convolution.

The entry flow consists of two layers of regular convolutions and three block structures. Each block has three depthwise separable convolution layers and residual connection channels are added. The residual structure is shown in Figure 4. Each depthwise separable convolution is composed of depthwise convolution and pointwise convolution. The channel separation calculation is first performed using 3 × 3 convolution, followed by feature merging using 1 × 1 convolution. After each convolution, batch normalization is performed, and the network output is adjusted using the mean and standard deviation on small batches to accelerate the convergence speed of the network. The middle flow has a total of 16 units, and the structure of each unit is the same as the block structure in the entry flow, with the difference being the number of convolution kernels. There are two blocks in the exit flow, and the structure of block 2-1 is consistent with the previous block 1. There is no residual connection in block 2-2.

### 3.4. Dual-Branch Network Structure

**Figure 5:**
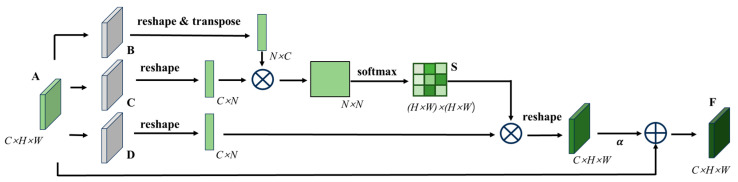
Position attention module.

The classic encoding decoding architecture network combines different scales of receptive field semantic information to improve the classification ability of the model, but such networks rely on downsampling to obtain advanced semantic information, ignoring spatial detail information. In order to segment the pallet stringer board from similar textures, the encoding region of this article adopts a dual-branch network structure, which processes spatial detail information and high-level semantic information respectively, achieving high-precision and efficient real-time segmentation of pallet components. The position attention module is shown in Figure 5. The position attention module can simulate rich contextual relationships between global features, thereby enhancing similar features at different positions and improving the semantic segmentation ability [29].

In the position attention module, local feature *A* from Figure 5 is first input into the convolutional layer to obtain feature matrices *B*, *C*, and *D*, $\left\{B,C,D\right\}\in R^{C\times H\times W}$. Then, we perform a series of operations such as reshaping, transposing, and multiplying matrices *B* and *C* to obtain *T*, and finally use the softmax layer to calculate the position attention $S\in R^{N\times N}$:

(1) $$S_{ji}=\frac{exp\left(B_{i}\cdot C_{j}\right)}{\sum_{i=1}^{N}exp\left(B_{i}\cdot C_{j}\right)}$$

where N=H×W represents the number of pixels; $B_{i}$ represents the *i*-th position element in matrix *B*; $C_{j\;}$ represents the j-th position element of matrix *C*; $S_{ij}$ represents the influence factor of the i-th position on the *j*-th position. Equation (1) indicates that the more similar the feature representations of two different positions are, the greater the correlation between them. At the same time, the generated feature matrix *D*$\in R^{C\times H\times W}$ is reshaped, and then multiplied with the position attention *S* matrix. The resulting result is reshaped again, multiplied with the scale parameter α, and summed with the feature map *A* to obtain the final output matrix $F\in R^{C\times H\times W}$:

(2) $$F_{j}=\alpha \sum_{i=1}^{N}\left(S_{ji}D_{i}\right)+A_{j}$$

where $D_{i}$ is the *i*-th element in matrix *D*; α is a learning parameter that gradually learns to allocate more weights from 0. Equation (2) shows that the result feature *F* for each position is the weighted sum of all position features and the original features. Therefore, the position attention module weights the features in each channel, allowing the model to focus on global contextual information and integrate broader contextual information into local features, promoting similar semantic features and improving the model’s semantic segmentation ability.

**Figure 6:**
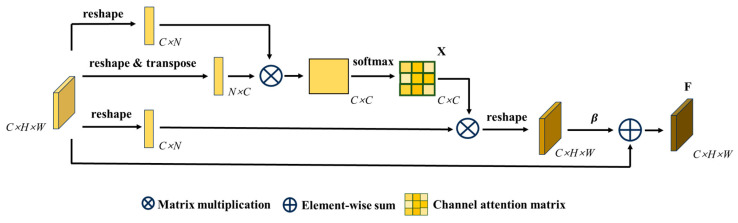
Channel attention module.

The channel attention module is shown in Figure 6. By utilizing the interrelationships between feature maps of different channels, it is possible to highlight the interrelated feature maps and promote specific semantic features. Therefore, it is necessary to explore the features of different channels [30].

In the channel attention module, the channel attention map *X* is directly calculated from the original feature matrix *A*. Firstly, the feature matrix is reshaped to obtain $A^{\prime }\in R^{C\times N},N=H\times W$. Then, $A^{\prime }$ prime is multiplied by its transpose matrix, and *X* is obtained through the softmax layer [31]:

(3) $$X_{ij}=\frac{exp\left[{A^{\prime }\times {\left(A^{\prime }\right)}^{T}}_{j}\right]}{\sum_{i=1}^{C}exp\left[{{A_{i}}^{\prime }\times {\left(A^{\prime }\right)}^{T}}_{j}\right]}$$

where ${A_{i}}^{\prime }$ represents the i-th position element value of the $A^{\prime }$ matrix, ${{\left(A^{\prime }\right)}^{T}}_{j}$ represents the j-th element value of the ${A_{i}}^{\prime }$ transpose matrix, and $X_{ij}$ represents the influence factor of the *i*-th channel on the *j*-th channel. Then, we transpose *X* and multiply it by the $A^{\prime }$ matrix, reshape the result, multiply it with the training parameter β, and add it to the matrix *A* to obtain the final result $F\in R^{C\times H\times W}$:

(4) $$F_{j}=\beta \sum_{i=1}^{C}\left(x_{ij}A_{i}\right)+A_{j}$$

where β is the training parameter, gradually learning weights from 0. From Equation (4), it can be concluded that the final feature of each channel is the weighted sum of all channel features and the original channel features. Therefore, the channel attention module can use the interdependence between channel graphs to enhance the feature representation of specific semantics.

### 3.5. Focal Loss Function

The characteristics of the pallet stringer board are similar to the other surfaces of the pallet, and there are a large number of other surfaces occupying pixels in the dataset. The detection accuracy is generally lower than that of medium and large target samples, and there are many problems such as missed judgments and false detections when detecting samples. To address the issue of class imbalance and improve model focus on small or difficult-to-detect regions during training, this paper adopts focal loss as the segmentation network’s loss function [32].

Focal loss dynamically adjusts the loss contribution of each sample by down-weighting the loss for well-classified examples, allowing the model to focus more on hard-to-detect regions, such as pallet slots that may be partially occluded or located at extreme angles. This characteristic makes it particularly suitable for our task, as precise slot localization directly impacts the AGV’s accuracy in pallet handling. To solve the problem of insufficient sample learning during model training, this paper uses focal loss as the loss function of the segmentation network.

(5) $$p_{t}=\left\{\begin{array}{ll} \hat{p} & if\;y=1\\ 1-\hat{p} & otherwise \end{array}\right.$$

(6) $$FL\left(p_{t}\right)=-\alpha_{t}{\left(1-p_{t}\right)}^{\gamma }\log\left(p_{t}\right)$$

The $p_{t}$ in Equation (5) reflects the degree of closeness to label *y*, and the larger the $p_{t}$ value, the closer it is to category *y*; ${\left(1-p_{t}\right)}^{\gamma }$ is the modulation factor. The more accurate the classification, the closer $p_{t}$ approaches 1, and the closer the modulation factor approaches 0. The $p_{t}$ also reflects the difficulty of classification. The larger the $p_{t}$, the higher the confidence level of classification, indicating that the sample is easier to classify. The smaller the $p_{t}$, the lower the confidence level of classification, indicating that the sample is more difficult to classify. In Formula (6), $\alpha_{t}$ is the weighting factor, which can suppress the imbalance in the number of positive and negative samples. By using γ, the imbalance in the number of simple and difficult-to-distinguish samples can be controlled.

## 4. Ablation Experiment and Analysis

### 4.1. Datasets and Evaluation Criteria

#### 4.1.1. Datasets Preparation and Processing

**Figure 7:**
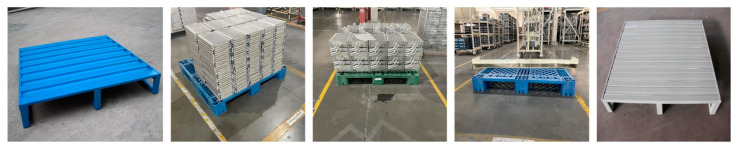
Partial of the pallet dataset.

The dataset used in this paper consists of images of pallets collected from warehouse scenarios. This dataset includes commonly used pallets in handling scenes, as shown in Figure 7, such as universal flat pallets for intermodal transport and steel flat pallets for railway freight. The dataset is categorized into single-type and multi-type pallet datasets.

**Figure 8:**
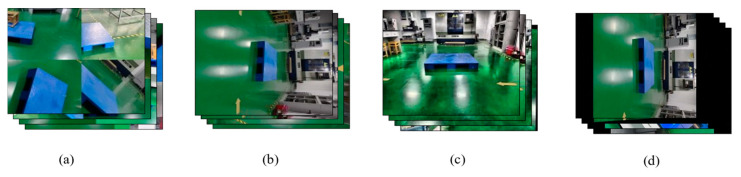
Data enhancement methods: (**a**) stitching, (**b**) rotation, (**c**) noise addition, and (**d**) cropping.

We label the pallet dataset by dividing it into three categories: background, stringer board, and pallet slots. In this study, the pallet dataset was constructed using the PASCAL VOC dataset format and annotated based on JSON annotation files. In this format, background pixels are represented as 0, the pallet stringer board is denoted by 1, and the pallet slots area is denoted by 2. The original pallet dataset consists of 500 images, covering commonly used standard pallet types, such as universal flat pallets for intermodal transport and steel flat pallets for railway freight. Due to the small dataset size, which may lead to overfitting and could affect the model’s generalization ability, data augmentation techniques such as rotation, cropping, noise addition, and stitching were applied only to the training set, expanding it to 3000 images. The augmentation methods for the original images are shown in Figure 8. After preprocessing, we manually filtered out images without targets that were generated during the data augmentation process. As the original images vary in resolution, all images were resized to 512 × 512 pixels. These augmented images enable the model to better adapt to various real-world scenarios, thereby improving training effectiveness and ultimately enhancing performance.

#### 4.1.2. Performance Evaluation

In order to verify the effectiveness and stability of the improved model, this study used the average intersection-to-union (*mIoU*) ratio and average pixel accuracy (*mPA*) as indicators to evaluate the network segmentation performance. The confusion matrix is used to evaluate the classification performance of the model. The four elements in the confusion matrix are as follows: true positive (*TP*), false positive (*FP*), true negative (*TN*), and false negative (*FN*).

Mean intersection union ratio (*mIoU*): The ratio between the intersection, the union of predicted results, and true values for each category is summed and then averaged to measure the segmentation accuracy of the model.

(7) $$mIoU=\frac{1}{M}\sum_{i=1}^{M}\frac{TP}{TP+FN+FP}$$

Mean pixel accuracy (*mPA*): The ratio of the number of correctly recognized pixels in each category to the total number of pixels in that category is summed and then averaged to measure the model’s pixel classification accuracy for each category.

(8) $$mPA=\frac{TP+TN}{TP+TN+FP+FN}$$

Among them, *TP* is the number of correctly predicted target image pixels by the network, *FP* is the number of incorrectly predicted target image pixels by the network, *TN* is the number of correctly predicted background pixels by the network, and *FN* is the number of incorrectly predicted background pixels by the network.

#### 4.1.3. Implementation Details

The LCS-Net proposed in this paper is trained end-to-end. It accurately identifies the stringer board and pallet slots of pallets at the pixel level, laying a solid technical foundation for achieving better localization results subsequently. All designed experiments were conducted on a Windows 10 operating system. The server environment used in the experiment included an Intel (R) Core (TM) i9-13900HX CPU, an NVIDIA GeForce RTX 4060 GPU, with 8 GB of graphics memory, and was configured with Python 3.9 + Python 2.1.2 + Cuda12.1. The input images to the model were cropped to a size of 512 × 512 pixels. The training objective was to minimize the loss function.

During training, the batch size was set to 8, and the gradient descent algorithm solved the minimum value by descending along the gradient. Due to the large number of model parameters, using the gradient descent algorithm can lead to excessive computational load. The random gradient descent algorithm can reduce the computational load, but the convergence speed is slow and easily affected by noisy samples, leading to the model falling into local optima. Therefore, the Adam algorithm was used during the training process to adaptively adjust the learning rate according to the gradient size and converge to the optimal solution faster [33].

For model training, the dataset was split into 80% for training, 10% for validation, and 10% for testing. The input images were resized to 512 × 512 pixels. The training objective was to minimize the loss function. The model was trained for 100 epochs. In addition, for the Adam optimizer when applied, hyperparameters β1 and β2 were set to 0.9 and 0.999. To optimize the model’s performance, the Adam optimizer adaptively adjusted the learning rate and ensured faster convergence. Early stopping was also employed during training to prevent overfitting, with training halted if the validation loss did not improve after a certain number of consecutive epochs. For the loss function, focal loss was employed to address the issue of class imbalance. $\alpha_{t}$ is a weighting factor set to 1 to balance the class imbalance. γ is the focusing parameter, set to 1 to focus more on harder-to-classify samples. This setup helps reduce the effect of easily classified examples, thereby emphasizing the harder ones during training, especially for class imbalance problems.

### 4.2. Ablation Studies

#### 4.2.1. Backbone Network Ablation Experiment

To verify the impact of the data augmentation methods employed in this study on the segmentation model’s generalization ability, the segmentation network proposed in this paper was trained on both the original data and the data augmented with the methods described. The training results are shown in Table 1.

The experimental results show that by enhancing and expanding the original dataset, all indicators on the test dataset improved. The intersection ratio of the stringer board increased by 12%, the intersection ratio of the pallet slots increased by 16%, and the average intersection ratio increased by 9.12%.

To verify the effectiveness of the improved backbone network, MobileNetv2 [34], Xception [35], and the proposed backbone network were used as the backbone networks of the LCS-Net proposed in this paper. Ablation experiments were conducted to verify the performance of the model. The evaluation indicators include the average intersection-to-union (*mIoU*) ratio and average frame rate (FPS). To ensure the reliability of the experimental results, each network was trained with 100 epochs under the same dataset and experimental environment. The training results are shown in Table 2.

From Table 2, it can be seen that the improved Xception backbone network proposed in this paper has an average intersection-to-union ratio that is 3.09% higher than the original Xception in the DeepLabv3+ network compared to the *mIoU*. When using the improved Xception proposed in this article as the backbone network, the processing speed is also faster compared to MobileNetv2. Therefore, considering both the processing speed and accuracy of the model, the backbone network proposed in this paper is the optimal choice.

#### 4.2.2. Evaluating the Impact of Attention Mechanisms

To further validate the choice of the position attention and channel attention modules in LCS-Net, we added a comparative experiment by replacing these modules. The spatial attention module and channel attention module effectively capture the feature dependencies in the spatial and channel dimensions, which is crucial for accurately identifying the pallet slot areas. The spatial attention module enhances the network’s focus on spatial relationships, aiding in more precise pallet slot localization, while the channel attention module highlights key feature maps, improving the network’s ability to distinguish between pallets and background noise. To further validate the role of the spatial attention module and channel attention module in the pallet segmentation task, an ablation study is designed as follows:

In Table 3, Backbone specifies the backbone network used for each model. All models in the experiments utilize the Xception backbone to ensure consistency in the architecture. Baseline indicates the model without any attention mechanism, Channel-Only indicates the model using only the channel attention module, Position-Only indicates the model using only the position attention module, and CBAM means that the variant applies the CBAM, which combines both channel and spatial attention mechanisms. ‘Position and Channel’ refers to the complete model that uses both the channel and position attention mechanisms.

As shown in Table 3, the ablation study evaluates the impact of different attention mechanisms on segmentation performance. The results indicate that attention mechanisms effectively improve segmentation accuracy, although different approaches lead to varying trade-offs in computational efficiency. Furthermore, the experimental results demonstrate that the proposed dual-attention mechanism, which combines position and channel attention, achieves the best performance, providing optimal segmentation accuracy while maintaining high inference speed.

The ablation experiment results show that the proposed attention mechanism combination in this paper has significant advantages in both segmentation accuracy and processing speed, meeting the real-time segmentation requirements of AGVs.

#### 4.2.3. Single-Type Pallet Comparative Experiment

In order to verify the advantages of the LCS-Net proposed in this article, compared to existing classical segmentation models, the classical segmentation network U-Net, PSPNet, DeepLabv3+, Fast-SCNN, and SegFormer. U-Net employs an encoder–decoder architecture, which effectively captures local features and performs excellently in medical image segmentation tasks, especially for small-sample datasets. PSPNet introduces a pyramid pooling module, which captures contextual information at different scales, making it highly suitable for segmenting images with multi-scale objects and enhancing global semantic information. DeepLabv3+ is based on deep dilated convolutions, effectively expanding the receptive field, and incorporates an encoder–decoder structure, making it ideal for high-precision segmentation tasks in complex scenes. Fast-SCNN is a lightweight network, specifically designed for real-time applications. It delivers good segmentation results while ensuring high inference speed, making it suitable for deployment in resource-constrained environments. SegFormer is based on a Transformer architecture, capable of handling multi-scale features and contextual information, and exhibits strong segmentation capabilities, particularly in complex backgrounds.

**Figure 9:**
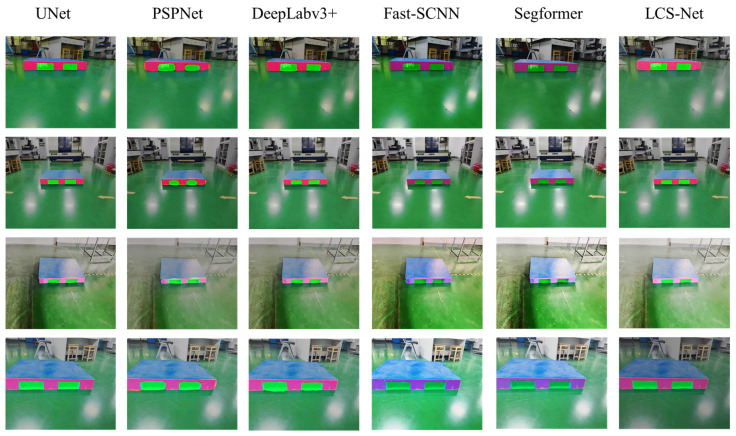
The segmentation effect of different models on pallets.

According to Table 4, the proposed model achieves the highest *mIoU* of 88.16%, surpassing all other models. The results indicate that the proposed model not only improves segmentation accuracy but also ensures high computational efficiency, making it well-suited for real-time applications. The segmentation effect of each network is shown in Figure 9.

From Figure 9, it can be seen that all six segmentation networks can effectively extract the pallet stringer board and pallet slots, but there are differences in the morphology of the extracted areas. The LCS-Net proposed in this article can accurately segment the pallet stringer board and the edge of the socket, effectively reducing edge burrs and false detections. At the same time, it significantly shortens the single image inference time. Compared with classical segmentation networks, it can better balance segmentation accuracy and speed.

**Figure 10:**
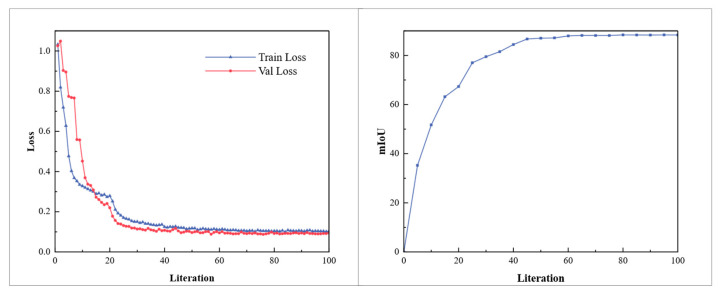
Loss curve and accuracy over iterations measured by the mean intersection over union under single-type pallet.

To validate and assess the performance of the segmentation network, a validation set is used during training to calculate the segmentation network loss. This ensures that the detector maintains overall performance throughout training, preventing overfitting and preserving generalization capability. The training results are shown in Figure 10, indicating that the segmentation model proposed in this paper can detect most pallet objects with high precision.

#### 4.2.4. Multi-Type Pallet Comparative Experiment

In human–robot hybrid intelligent warehouses, various types of pallets frequently appear, with differing pallet slots sizes and center distances. Therefore, the network must have a certain degree of generalization ability to accurately locate the centers of the pallet slots. This study aims to verify the superiority of the segmentation model proposed herein over existing classical segmentation models in detecting multiple types of pallets. To achieve this, we conducted comparative experiments using the classic segmentation network U-Net, PSPNet, DeepLabv3+, Fast-SCNN, SegFormer, and the improved model proposed in this paper. After 100 epochs of training, we obtained evaluation metrics for the models on various datasets containing different types of pallets, as shown in Table 5.

**Figure 11:**
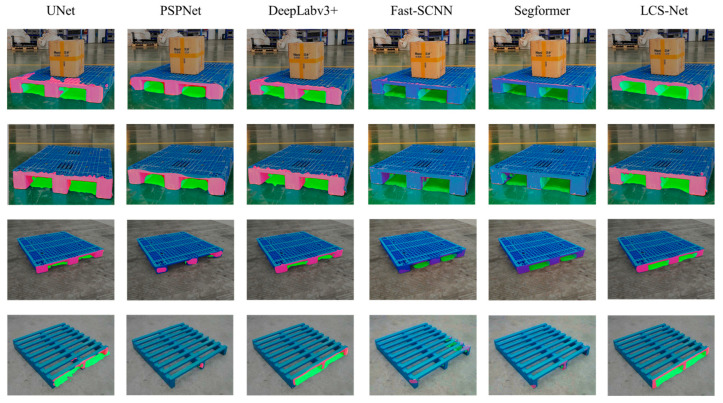
Segmentation results of different models on different pallet types.

From Table 5, the results demonstrate that our model performs competitively in terms of segmentation accuracy, with an *mIoU* of 78.02%. It achieves excellent performance in pallet slot segmentation, comparable to the highest-performing models. While SegFormer leads in segmentation accuracy, our model offers a better balance between segmentation performance and inference speed. Fast-SCNN is the fastest model, but its segmentation accuracy is lower than that of others. Compared to other classical networks, our model achieves a balance between segmentation accuracy and speed. The segmentation results of our network on different types of pallets are illustrated in Figure 11.

From Figure 11, it can be observed that the LCS-Net accurately segments the pillar surfaces and plug-hole edges of various types of pallets. This model effectively reduces edge artifacts and false detection occurrences. Moreover, it significantly reduces the inference time per image compared to classic segmentation networks. Thus, it achieves a good balance between segmentation accuracy and speed.

**Figure 12:**
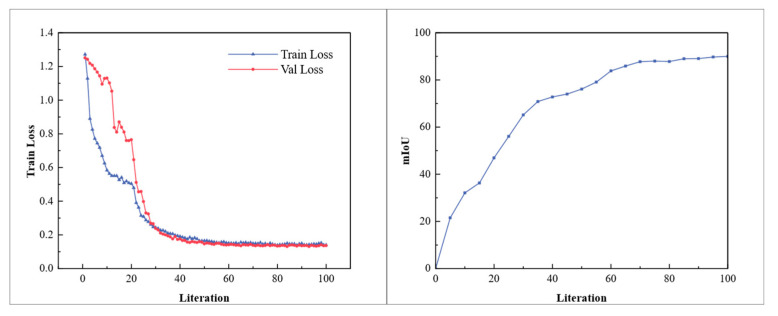
Loss curve and accuracy over iterations measured by mean intersection over union under multi-type pallet.

To validate and assess the performance of the segmentation network, we use a validation set to calculate the segmentation network loss. This ensures that the detector maintains overall performance throughout training, preventing overfitting and preserving generalization capability. The training results are shown in Figure 12, indicating that the segmentation model proposed in this paper can detect most pallet objects with high precision.

### 4.3. Location of Pallet Based on LCS-Net

#### 4.3.1. Positioning the Center Point of the Pallet Stringer Board

To achieve the positioning of the center point of the pallet stringer board, it is necessary to first calibrate the extrinsic parameters of the camera in the warehouse working environment. This will determine the transformation relationship between the pixel coordinate system and the pallet stringer board coordinate system, generating a conversion formula between the two coordinate systems. By processing the image, the minimum enclosing rectangle of the segmented pallet stringer board can be obtained, from which the pixel coordinates of the center of the stringer board are derived. Using the coordinate transformation formula, the three-dimensional coordinates of the center point of the pallet stringer board can be obtained.

To obtain the transformation parameters between the pixel coordinate system and the pallet coordinate system, this paper uses Zhang’s calibration method to calibrate the camera. By using the Calibration Toolbox, the camera’s intrinsic parameters, including the effective focal length and lens distortion coefficients, are obtained. The distortion coefficients are used to correct image distortion caused by lens distortion. The camera’s extrinsic matrix is calibrated using a chessboard calibration plate, resulting in the camera’s extrinsic matrix [*R_PalletToCamera_*|*T_PalletToCamera_*]. Based on the pinhole imaging model and the obtained camera extrinsic and intrinsic parameters, the transformation relationship between the pixel coordinate system and the pallet coordinate system is established. The position of the center of the pallet stringer board in the pallet coordinate system can be expressed as $\left(X,Y,Z\right)$. The transformation relationship between the position of a point on the pallet stringer board in the pallet coordinate system and its projected point coordinates is given by the following:

(9) $$\left(\begin{array}{l} X\\ Y\\ Z\\ 1 \end{array}\right)=Z_{C}{\left(\begin{array}{cc} R_{PalletToCamera} & {\;T}_{PalletToCamera}\\ 0 & 1 \end{array}\right)}^{-1}\left(\begin{array}{llll} \frac{f}{d_{x}} & 0 & u_{0} & 0\\ 0 & \frac{f}{d_{y}} & v_{0} & 0\\ 0 & 0 & 1 & 0 \end{array}\right)\left(\begin{array}{l} u\\ v\\ 1 \end{array}\right)$$

where f is the effective focal length, $d_{x}$ and $d_{y}$ represent the physical length of a pixel on the camera sensor in the x and y directions, respectively, and $u_{0}$ and $v_{0}$ represent the coordinates of the center of the camera sensor in the pixel coordinate system.

In actual warehouse scenarios, it is necessary to accurately locate the centroid of the pallet stringer board to ensure accuracy and stability during handling. The center of the pallet stringer board can be determined by finding the center of the minimum enclosing rectangle. By using the segmented mask of the pallet stringer board, the rotating caliper method is applied to iteratively compute the minimum enclosing rectangle of the contour point set for each target instance mask. First, the convex hull algorithm is used to obtain the convex hull E of the stringer board contour. Based on the convex hull, the minimum area enclosing the rectangle is found. By comparing the areas of the enclosing rectangles, the one with the smallest area is selected as the minimum enclosing rectangle of the stringer board contour. During the enumeration process, the top-left vertex (*x*_1_, *y*_1_), length *l*, and width *w* of the enclosing rectangle are recorded. The pixel center coordinates O are defined as $\left(x_{1}+\frac{l}{2},y_{1}+\frac{w}{2}\right)$. After obtaining the pixel center of the pallet stringer board, the coordinate transformation formula is used to convert the pixel coordinates of the stringer board into the three-dimensional coordinate information (*X*, *Y*, *Z*) of the center point of the pallet stringer board.

#### 4.3.2. Evaluation Experiment of Pallet-Positioning Accuracy Under Different Distance

**Figure 13:**
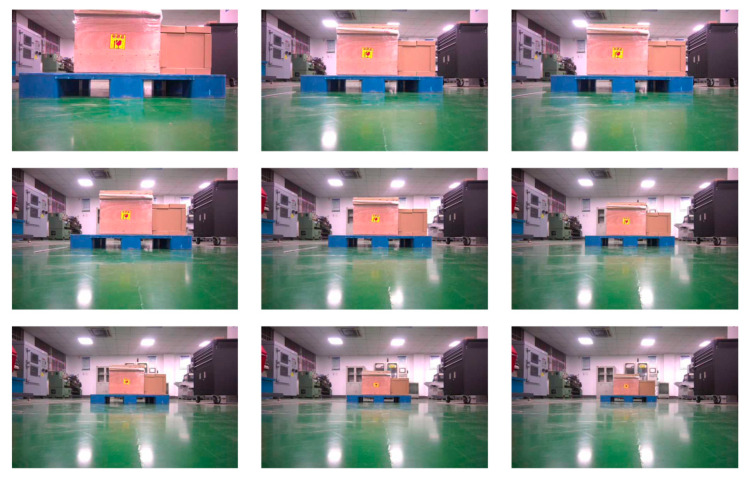
Experiments on pallet positioning at different distances.

The equipment used in this experiment is shown in Figure 13. The experiment varied the distance and angle between the camera and the pallet. The distance was adjusted between 1 m and 3 m, with specific distances set at 1 m, 1.25 m, 1.5 m, 1.75 m, 2 m, 2.25 m, 2.5 m, 2.75 m, and 3 m, to test the pallet-positioning accuracy at different distances.

#### 4.3.3. Evaluation Experiment of Pallet-Positioning Accuracy Under Different Offset Angles

**Figure 14:**
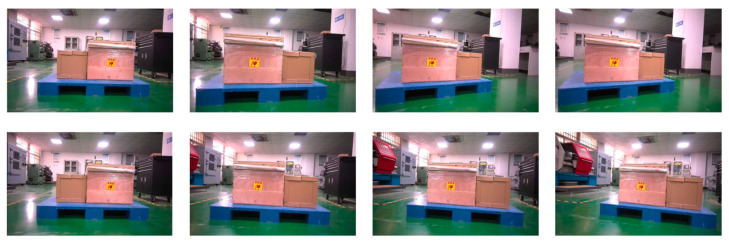
Experiments on pallet positioning at different angles.

Considering that the pallet’s position may not be fixed in human–robot hybrid scenarios, different pallet offset angles were selected as experimental variables. The pallet was placed 1.5 m away from the camera, and the pallet’s offset angle was adjusted in 10° increments within a range from −30° to 30°. The experimental setup is illustrated in Figure 14.

#### 4.3.4. Evaluation Experiment Result Analysis

The distance between the camera and the pallet is the variable in this experiment and is denoted by z. The variable x represents the horizontal relative displacement based on the pallet, and y represents the vertical relative distance between the camera and the pallet. The absolute errors at different distances, the mean absolute error (MAE), and the standard deviations (STDs) are shown in Table 6.

The results indicate that at various experimental distances, the maximum absolute errors for horizontal displacement, vertical displacement, distance, and angle are 9.19 mm 7.59 mm, 12.15 mm, and 1.68°, respectively. The minimum absolute errors are 1.45 mm, 1.42 mm, 0.95 mm, and 0.43°.

The angle between the camera and the pallet is the variable in this experiment and is denoted by β. The variable x represents the horizontal relative displacement based on the pallet, and *y* represents the vertical relative distance between the camera and the pallet. The results are shown in Table 7.

The results indicate that at various experimental distances, the maximum absolute errors for horizontal displacement, vertical displacement, distance, and angle are 3.78 mm, 5.78 mm, 6.54 mm, and 2.08°, respectively. The minimum absolute errors are 0.93 mm, 0.52 mm, 1.76 mm, and 0.79°. The experimental results indicate that the proposed pallet-positioning algorithm can recognize and locate pallets within a 30-degree deviation angle, with an average absolute error of within 1 cm for distance and 3° for angle.

### 4.4. Validation of Generalization Ability Across Different Environments

#### 4.4.1. Illumination and Noise Joint Interference Experiment

**Figure 15:**
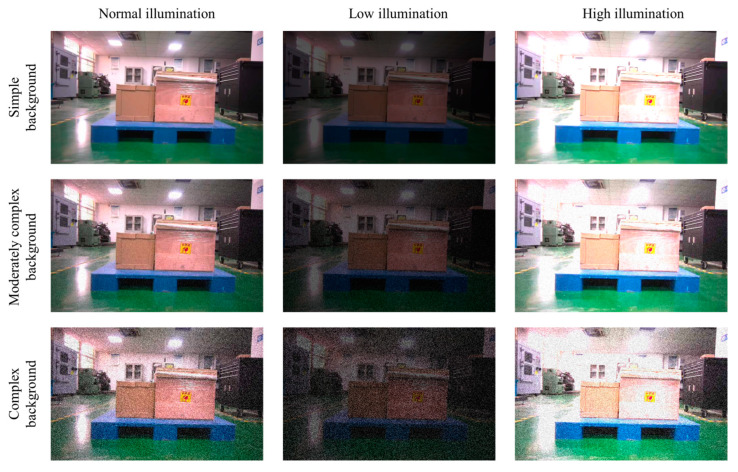
Hybrid experiment at a distance of 1 m.

Building upon the original distance and angle variation experiments, this section further investigates the impact of illumination changes and background noise on the pallet localization accuracy of the LCS-Net model to comprehensively evaluate its robustness. The experimental variable includes different illumination intensities and background complexities. Normal illumination simulates a standard indoor warehouse environment, low illumination simulates nighttime or insufficient lighting conditions, and high illumination simulates direct sunlight or light reflections. Three noise levels (simple, moderately complex, and complex) are set by adjusting noise parameters to simulate three warehouse scenarios: simple background, moderately complex background, and complex background. To obtain more realistic robustness data, the interaction effects of illumination and background noise are comprehensively considered, generating nine combination groups. Fix the pallet at a distance of 1 m and an angle of 0°, gradually change the lighting intensity and background complexity, capture images, and use LCS-Net to segment the pallet position, as shown in Figure 15.

#### 4.4.2. Different Environments Result Analysis

The angle between the camera and the pallet is the variable in this experiment, denoted by β. The variable *x* represents the horizontal relative displacement based on the pallet, and *y* represents the vertical relative distance between the camera and the pallet. The results are shown in Table 8.

The table shows the absolute errors in the *x*, *y*, and *z* directions and the angle β under different lighting conditions and background complexities. Under normal illumination and simple background and low illumination and complex background, the maximum error is far below 10mm. Under normal illumination, the maximum error is 1.20 mm, and under low lighting and a complex background, the maximum error is 1.70 mm. The maximum angle error is 1.10°.

Even under challenging conditions such as low illumination and complex backgrounds, the maximum errors remain within the acceptable range for AGV operations, ensuring reliable positioning performance. LCS-Net’s robustness and stability guarantee consistent performance in practical applications, making it suitable for AGV pallet positioning in various environments.

## 5. Conclusions

We present a component segmentation network, LCS-Net, to address the precise positioning of multiple types of pallets, which uses a dual-branch attention mechanism to obtain spatial detail information and optimize the weights of deep features to solve the problem of inaccurate edge segmentation of pallets. The position attention module selectively aggregates the features of each position by weighting and summing up the features of all positions; The channel attention module obtains the interdependence between different feature channels, strengthens the learning of important channel features, and improves the semantic segmentation effect. Adding the outputs of two attention modules can further improve feature representation and enhance segmentation performance.

Test results indicate that our model outperforms existing segmentation methods in terms of average accuracy, processing speed, and generalization capability. The LCS-Net we proposed achieves an *mIoU* of 88.16% on the single-class pallet dataset, representing a 4.89% improvement over the classic segmentation networks DeepLabv3+. On the multi-class pallet dataset, the *mIoU* is improved by 10.11% compared to the classic segmentation network DeepLabv3+, and the average pixel accuracy is increased by 10.41%. We conducted a visual localization experiment for pallet picking, where AGV was guided by a camera to perform picking tasks for pallets placed in unknown locations. These localization experiments were conducted at various distances and angles. The results show that the LCS-Net-based visual localization method can effectively recognize and locate multi-category pallet targets in the warehousing field. Moreover, in experiments considering interference factors such as illumination changes and background noise, LCS-Net still maintains high accuracy, further proving the model’s stability and robustness in complex environments. Compared to using specialized data sources like depth data for pallet localization, our proposed pallet localization algorithm meets the accuracy and speed requirements necessary for practical production tasks.

Although this study focuses on pallet positioning in intelligent warehousing scenarios, the system can be easily transplanted to other scenarios by changing the dataset and setting corresponding loss functions. It can achieve precise detection and positioning of randomly placed objects in human–robot hybrid intelligent warehouse scenarios using component segmentation, such as robots, empowering new industries and providing technical support for highly intelligent related fields.

## Figures and Tables

**Table 1:** Comparison between raw data and expanded data.

Data Type	Stringer Board IoU/%	Pallet Slots IoU/%	*mIoU*/%
Raw data	64	59	74.15
Enhanced data	76	75	83.27

**Table 2:** Experimental results of different backbone networks.

Backbone	Model	*mIoU*/%	FPS
MobileNetv2	DeepLabv3+	84.36	36.88
Xception	DeepLabv3+	83.27	32.73
Ours	DeepLabv3+	86.36	43.23

**Table 3:** Performance comparison of LCS-Net with different attention mechanisms.

Backbone	Attention Mechanism	*mIoU*/%	FPS
Xception	Baseline	69.09	45.71
Xception	Channel-Only	75.95	43.89
Xception	Position-Only	73.48	44.25
Xception	CBAM	83.99	37.83
Xception	Position and Channel	87.37	43.57

**Table 4:** Comparison of network evaluation indicators under single-type pallet.

Method	*mIoU*/%	Stringer Board IoU/%	Pallet Slots IoU/%	*mPA*/%	FPS
U-Net	77.87	66	69	96.49	34.89
PSPNet	72.09	64	67	87.59	45.88
DeepLabv3+	83.27	76	75	90.12	32.73
Fast-SCNN	66.45	66	67	79.17	47.55
SegFormer	82.88	82	83	90.48	28.06
Ours	88.16	82	83	93.82	49.39

**Table 5:** Comparison of network evaluation indicators under multi-type pallet.

Method	*mIoU*/%	Stringer Board IoU/%	Pallet Slots IoU/%	*mPA*/%	FPS
U-Net	65.28	45	59	73.48	34.27
PSPNet	70.01	55	58	76.79	41.04
Deeplabv3+	67.91	46	60	74.23	29.96
Fast-SCNN	66.45	66	67	79.17	43.43
SegFormer	78.44	72	73	83.79	26.84
Ours	78.02	63	73	84.64	39.79

**Table 6:** The absolute error, mean absolute error, and standard deviation of the camera and pallet at different distances.

*z*/mm	Absolute Visual Positioning Error			
	*x*/mm	*y*/mm	z/mm	β/°
1000	2.76	1.42	0.95	0.57
1250	1.45	2.65	2.75	0.43
1500	1.87	4.11	4.81	0.97
1750	2.66	3.67	6.29	0.96
2000	3.98	4.74	3.79	1.15
2250	5.86	5.48	5.24	1.68
2500	4.86	7.59	9.43	1.18
2750	6.54	6.41	8.31	1.09
3000	9.19	7.51	12.15	0.51
MAE	4.35	5.02	5.75	0.86
STD	2.38	1.64	2.90	0.37

**Table 7:** The absolute error, mean absolute error, and standard deviation at different pallet offset angles.

β/°	Absolute Visual Positioning Error			
	*x*/mm	*y*/mm	*z*/mm	β/°
−30	0.93	0.52	1.76	1.67
−20	1.22	1.23	3.43	1.54
−10	1.46	1.21	3.96	1.09
0	1.08	4.89	3.22	0.79
10	2.78	3.76	4.98	1.98
20	1.88	5.27	4.17	2.08
30	3.78	5.78	6.54	1.81
MAE	1.73	3.88	3.86	1.57
STD	0.98	1.97	1.17	0.41

**Table 8:** The absolute error, mean absolute error, and standard deviation in different environments.

Illumination	Background	Absolute Visual Positioning Error			
		*x*/mm	*y*/mm	*z*/mm	β/°
Normal	Simple	2.76	1.42	0.95	0.57
Normal	Moderately complex	2.98	2.53	1.04	0.65
Normal	Complex	3.45	2.67	1.20	0.72
Low	Simple	4.20	3.17	1.35	0.85
Low	Moderately complex	4.51	3.52	1.67	0.96
Low	Complex	5.10	4.27	1.70	1.10
High	Simple	3.10	1.51	1.10	0.65
High	Moderately complex	3.78	2.73	1.27	0.71
High	Complex	3.97	3.10	1.36	0.75
MAE		3.76	2.77	1.29	0.77
STD		0.73	0.85	0.25	0.16

## Data Availability

Data will be made available upon request.
